# Intestinal colonization regulates systemic anti-commensal immune sensitivity and hyperreactivity

**DOI:** 10.3389/fimmu.2023.1030395

**Published:** 2023-05-22

**Authors:** Regula Burkhard, Mia Koegler, Kirsty Brown, Kirsten Wilson, Lukas F. Mager, Amanda Z. Zucoloto, Carolyn Thomson, Roopa Hebbandi Nanjundappa, Isla Skalosky, Shokouh Ahmadi, Braedon McDonald, Markus B. Geuking

**Affiliations:** ^1^ Department of Microbiology, Immunology and Infectious Diseases, Cumming School of Medicine, University of Calgary, Calgary, AB, Canada; ^2^ Department of Physiology and Pharmacology, Cumming School of Medicine, University of Calgary, Calgary, AB, Canada; ^3^ Snyder Institute of Chronic Diseases, Cumming School of Medicine, University of Calgary, Calgary, AB, Canada; ^4^ Immunology Research Group, Cumming School of Medicine, University of Calgary, Calgary, AB, Canada; ^5^ Department of Critical Care Medicine, Cumming School of Medicine, University of Calgary, Calgary, AB, Canada

**Keywords:** commensal *E. coli*, immunoglobulins, microbiota, immunogenicity, metabolism

## Abstract

Healthy host-microbial mutualism with our intestinal microbiota relies to a large degree on compartmentalization and careful regulation of adaptive mucosal and systemic anti-microbial immune responses. However, commensal intestinal bacteria are never exclusively or permanently restricted to the intestinal lumen and regularly reach the systemic circulation. This results in various degrees of commensal bacteremia that needs to be appropriately dealt with by the systemic immune system. While most intestinal commensal bacteria, except for pathobionts or opportunistic pathogen, have evolved to be non-pathogenic, this does not mean that they are non-immunogenic. Mucosal immune adaptation is carefully controlled and regulated to avoid an inflammatory response, but the systemic immune system usually responds differently and more vigorously to systemic bacteremia. Here we show that germ-free mice have increased systemic immune sensitivity and display anti-commensal hyperreactivity in response to the addition of a single defined T helper cell epitope to the outer membrane porin C (OmpC) of a commensal *Escherichia coli* strain demonstrated by increased *E. coli*-specific T cell-dependent IgG responses following systemic priming. This increased systemic immune sensitivity was not observed in mice colonized with a defined microbiota at birth indicating that intestinal commensal colonization also regulates systemic, and not only mucosal, anti-commensal responses. The observed increased immunogenicity of the *E. coli* strain with the modified OmpC protein was not due to a loss of function and associated metabolic changes as a control *E. coli* strain without OmpC did not display increased immunogenicity.

## 1 Introduction

The commensal microbes that make up our intestinal microbiota have established a carefully tuned relationship with our immune system to allow for a peaceful co-existence. The microbiota modulates mucosal and systemic immune responses of the host. Microbial metabolites are key molecular mediators between the host and the microbiota (1). Local mucosal adaptive immune adaptations to intestinal microbial colonization include induction of secretory IgA as well as a carefully orchestrated CD4 T cell response consisting of Th1, Th17, and regulatory T cell (Treg) responses, for example (2). More recent work suggests that intestinal microbes, such as *Akkermansia muciniphila*, also induce systemic antibody responses even under homeostatic conditions with an intact intestinal barrier (3–5).

The systemic adaptive immune response towards commensal bacteria reaching systemic sites is different to local mucosal responses and mainly driven by antigen-specific Th1 and IgG (6). Systemic commensal bacteremia is rather common and can be caused by reduced intestinal barrier function as a result of infections (7), mucosal damage due to dental hygiene (8), non‐steroidal anti‐inflammatory drugs (9) or dietary fats (10, 11), to just mention a few examples. Several studies have demonstrated robust levels of systemic IgG with anti-commensal reactivity in healthy individuals with higher titers in patients with Crohn’s disease (12–17).

Most studies have investigated how microbial metabolites modulate the innate and adaptive immune response towards commensal bacteria residing and contained within the intestinal lumen (1). However, it is now evident that metabolites derived from the intestinal microbiota can also modulate systemic immune responses, for example against cancer cells (18–23).

Whether the intestinal commensal microbiota also modulates systemic anti-commensal immune responses when these microbes reach systemic sites has so far not been studied to the same degree. We therefore tested the hypothesis that intestinal colonization at birth regulates systemic anti-commensal hyperreactivity in adulthood. We developed and previously described a model to study commensal *Escherichia coli*-specific CD4 T cell responses using a genetically modified commensal *E. coli* strain expressing a single additional defined T helper neo-antigen (24). In this model, the MHCII I-A^b^-restricted epitope gp61, derived from the Lymphocytic Choriomeningitis Virus (LCMV) glycoprotein, was introduced into loop 7 of the outer membrane porin C (OmpC) of *E. coli*. To achieve this, we first generated an OmpC deletion mutant *E. coli ΔompC* and then inserted a modified *ompC* gene with the gp61 epitope inserted into loop 7 to generate *E. coli ΔompC::ompC_gp61* (hereafter referred to as *E. coli ompC_gp61* for simplicity) (24).

We have previously demonstrated that the modified OmpC protein is processed following intravenous injection of live *E. coli* and the gp61 neo-epitope is presented on the MHC II I-A^b^ molecule to gp61-specific T cells (24). In this study, we investigated whether the systemic immune system is sensitive enough to recognize this addition of a single additional T helper cell epitope and how this is modulated by the intestinal microbiota.

We found that the systemic immune system of germ-free mice displayed a higher sensitivity and was able to detect the addition of a single T cell epitope to OmpC resulting in increased systemic, but not local mucosal, immunogenicity reflected by increased *E. coli*-specific T cell-dependent IgG responses. This *E. coli*-specific hyperreactivity was only observed in germ-free female C57BL/6 mice and was abrogated when mice were colonized with a defined intestinal microbiota at birth indicating that intestinal colonization also modulated systemic anti-commensal reactivity. The term ‘hyperreactivity’ is used in this study as it relates to *E. coli*-specific IgG responses and does not refer to hyperreactivity observed in allergic hypersensitivity, for example.

Although we were not able to detect gp61-specific T cell responses by tetramer staining, the observed increased *E. coli*-specific IgG responses were T cell dependent and, importantly, were also MHC II haplotype-dependent. Increased *E. coli*-specific reactivity was only observed in germ-free C57BL/6 mice (I-A^b^) that are able to present the I-A^b^-restricted gp61 peptide but not in germ-free BALB/c mice (I-A^d^ and I-E^d^) that are not able to present the gp61 peptide, which provides indirect evidence for gp61-specific T cell-dependence. Furthermore, increased immunogenicity was not mediated by structural changes in the *E. coli* surface structure or changes in OmpC function. Increased immunogenicity was no longer observed in mice colonized with a defined microbiota indicating that intestinal colonization not only modulates local mucosal responses but also regulates systemic anti-commensal immune sensitivity and hyperreactivity.

## 2 Materials and methods

### 2.1 Mice

Adult 8 to 12-week-old C57BL/6, BALB/c and *TCRbd^-/-^* were bred and housed under germ-free or gnotobiotic conditions in flexible film isolators (NKP isotec) at the germ-free/gnotobiotic facility of the International Microbiome Centre (IMC), University of Calgary, Canada. sDMDMm2 gnotobiotic animals were generated as previously described (25, 26). Germ-free or colonization status was confirmed by a combination of fecal Gram-staining, Sytox green DNA staining, aerobic and anaerobic culture, and 16S rRNA gene amplicon sequencing. All experiments were performed using animal use protocols AC16-0235 and AC21-0075 approved by the University of Calgary Health Sciences Animal Care Committee (HSACC).

### 2.2 Bacterial culture conditions, intravenous injections and quantification

The bacterial strains used in this study are listed in **Table 1**. Various *E. coli* strains, *Citrobacter rodentium*, *Pseudomonas aeruginosa* and *Salmonella typhimurium* were grown aerobically in LB medium. *Akkermansia muciniphila* was grown in BHI under anaerobic conditions and *Klebsiella oxytoca* was aerobically cultured in tryptic soy broth.

**Table 1:** Bacterial strains.

Strain	Genotype description	Reference/Source
*Escherichia coli* MG1655	wildtype	(24)
OmpC‐deficient *E. coli*	*ΔompC::TetRA*	(24)
OmpC_gp61 *E. coli*	*ΔompC::ompC_gp61*	(24)
*Citrobacter rodentium* DBS100	wildtype	ATCC 51459
*Akkermansia muciniphila*	wildtype	ATCC BAA-835
*Klebsiella oxytoca*	wildtype	ATCC 68831
*Pseudomonas aeruginosa*	wildtype	In-house strain
*Salmonella typhimurium*	wildtype	ATCC SL1344
*E. coli* Xen14	wildtype	Perkin Elmer, Cat # 119223

For intravenous *E. coli* injections, a single colony was inoculated in 15ml LB media and grown overnight at 37°C with shaking at 220rpm. Bacteria were harvested by centrifugation, washed in sterile PBS and resuspended at a concentration of 5x10^7^ CFU/ml in PBS, 200µl were used for intravenous injections resulting in a final dose of 1x10^7^ CFU. All steps were performed aseptically under a sterile laminar flow hood. Peracetic acid inactivation was performed as described previously (27). Briefly, an aliquot of the respective *E. coli* strain was incubated in 10ml 0.4% peracetic acid for 1 hour at room temperature. The inactivated bacteria suspension was washed three times with sterile PBS and resuspended to a concentration of 5x10^7^ CFU/ml in PBS. 200µl was used for intravenous injections resulting in a final dose of 1x10^7^ CFU. Sterility of peracetic acid killed inoculum was confirmed by standard culture methods.

To assess bacterial load in the blood, 50µl blood was collected (from tail vein) in heparin tubes and plated on LB agar plates.

### 2.3 Bacterial flow cytometry to measure *E. coli*-specific Ig responses in serum

Bacterial flow cytometry of bacterial specific serum immunoglobulins was performed as previously described (28). Briefly, a single colony of plated bacteria was inoculated in 3ml of the appropriate growth media and cultured overnight at 37°C. Bacteria were washed twice in sterile filtered PBS/2%BSA/0.02% NaN_3_ before resuspending at a density of approximately 10^7^ CFU/ml. Serum samples were incubated at 56°C for 30 minutes to inactivate complement and serially diluted with staring dilutions at 1:10. 25µl of bacterial suspension (approx. 10^5^ bacteria per sample) were then incubated with diluted serum for 1 hour at 4°C. Samples were washed twice and incubated with secondary anti-IgG2b-BV786 (clone R12-3, BD Bioscience), anti-IgG2c-BV421 (clone RMG2a, Biolegend), and anti-IgM-PE (clone R6-60.2, BD Bioscience) over night. After washing, samples were resuspended in PBS/2%BSA/0.02% NaN_3_ containing SYTO BC (Thermo Fisher) and acquired on a FACS Canto flow cytometer (BD Biosciences) using FSC and SSC parameters in logarithmic mode and a low FSC and SSC threshold to allow bacterial detection. FACS data were analyzed using FlowJo software (TreeStar, USA) and the levels of bacteria-specific antibodies present in the samples were expressed as geometric mean fluorescent intensity (gMFI) against absolute immunoglobulin concentration in the sample (measurement of total immunoglobulin concentration is outlined below).

### 2.4 Measurement of bacterial IgA coating in feces by flow cytometry

IgA-coated fecal bacteria were measured by flow cytometry as previously described (29). Briefly, fecal material was collected from the SI and colon of mice. The weight of the fecal material was determined, and samples were reconstituted to 100mg/ml in sterile PBS. Fecal homogenates were centrifuged to remove large particles, and the supernatant was passed through a sterile 22µm sterile nylon filter. An aliquot of the bacterial suspension was washed in PBS/1%BSA, subsequently non-specific binding sites were blocked with PBS/1%BSA/20% rat serum for 20 minutes at 4°C. Bacteria were washed twice and incubated with secondary anti-IgA-BV605 (clone 10-3, BD Biosciences) for 1 hour at 4°C. After washing, samples were resuspended in PBS/1% BSA/Syto BC (Thermo Fisher) and data were acquired on a FACS Canto flow cytometer (BD Biosciences) using FSC and SSC parameters in logarithmic mode and a low FSC and SSC threshold to allow bacterial detection. FACS data were analyzed using FlowJo software (TreeStar, USA).

### 2.5 Flow cytometry

Spleens of *E. coli* primed or LCMV infected mice were collected 14 days post infection and filtered through a 70µm cell strainer to obtain a single cell suspension. Following red blood cell lysis, I-A^b^/gp61-PE Tetramer (NIH Tetramer Core Facility) staining was performed for 1h at 37°C in FACS buffer (PBS, 2% FCS, 2mM EDTA). Cells were washed and stained with fixable viability dye eFluor506 (eBioscience) and FcR-block (BD Biosciences) in PBS for 20 minutes at 4°C. Subsequently, cells were washed and stained for surface markers in FACS buffer for 20 minutes at 4°C, followed by fixing with 2% PFA. The following antibodies were used for the cell surface staining: CD45-BV421 (clone 30-F11, BD Biosciences), TCRb-BV786 (clone H57-597, BD Biosciences), CD4-BV605 (clone RM4-5, BD Biosciences) and CD8a-AF700 (clone 53-6.7, BD Biosciences). Data was acquired on a Sony SP6800 flow cytometer, and analyzed using FlowJo software (TreeStar).

### 2.6 LCMV infection

LCMV-Armstrong was kindly provided by Dr. Marc Horwitz and viral stocks were prepared by a single passage on BHK-21 cells. Mice were infected intravenously with 200 plaque forming units (PFU) of LCMV-Armstrong to generate an acute infection.

### 2.7 Immunoglobulin electrochemiluminescence immune assay

Total concentrations of different immunoglobulin isotypes in the serum were determined using the Mouse Isotyping Panel 1 kit according to the manufacturer’s protocol (Meso Scale Discovery).

### 2.8 Membrane integrity characterization

Overnight cultures or subcultured bacteria (grown to OD_600_ 0.5) were centrifuged, and washed twice with sterile PBS. Approximately 10^5^ bacteria per sample were labelled with SYTOX Green (Thermo Fisher) or SYTO BC (Thermo Fisher) nucleic acid stains for 5 minutes at room temperature and cells were subsequently analyzed by a BD Bioscience FACSCanto.

### 2.9 Metabolomics

Metabolites were extracted from the supernatant and pellet of liquid *E. coli* at exponential and lag growth phases. The different *E. coli* strains were grown overnight or subcultured to OD_600 = _0.5. Metabolites from supernatant were extracted in 100% methanol, precipitated at -20°C for 1 hour, centrifuged and diluted into a linear range (1:20 final dilution) with 50% methanol. To extract intracellular metabolites, bacterial pellets were snap-frozen in liquid nitrogen, resuspended in cell lysis buffer (1M Tris, 5M NaCl, 0.5M EDTA, 10% SDS in H_2_O) and sonicated. Samples were subjected to untargeted metabolomics analysis using LC-MS. Metabolomics data were acquired at the Calgary Metabolomics Research Facility. Samples were centrifuged (max. speed, 15 minutes, 4°C), then 200µL was transferred to a deep-well 96-well plate (Thermo Fisher). Further processing was performed at the Calgary Metabolomics Research Facility (CMRF). Briefly, metabolites were separated using Ultra High-Performance Liquid Chromatography (UHPLC) Mass Spectrometry (MS). A hydrophilic interaction liquid chromatography column was used as the stationary phase (Syncronic HILIC, Thermo Fisher) and the mobile phase was comprised of a gradient of Solvent A (20mM ammonium formate pH 3 in H_2_O) and B (MS Grade Acetonitrile with 0.1% formic acid). MS data were collected on a Q Exactive HF Hybrid Quadrupole-Orbitrap Mass Spectrometer using negative-mode electrospray ionization. For targeted analysis, metabolites were identified using retention times and MS profiles of a library of in-house verified standards using MAVEN freeware. Relative abundance was estimated based on area under the curve of spectral profiles. Downstream analysis was performed in the RStudio environment (30) using R programming language (31). Data manipulation was performed using the dplyr package (32). For principal coordinates analysis, Durbin scaling was applied, and analyses were performed using functions from the vegan package (33). For differential abundance analysis, data were normalized to the average of the WT samples. Plots were generated using the ggplot (34) and pheatmap (35) packages.

### 2.10 Statistical analyses

Biological replicates consisted of separate individual mice. Normally-distributed data are show as mean ± standard error of the mean (SEM), and tested using unpaired T-test (to compare 2 groups) or one-way ANOVA followed by Tukey’s *post-hoc* (for >2 groups). Non-normally distributed data is shown as median ± interquartile range and tested using Mann-Whitney U test (for 2 groups), or Kruskal-Wallis followed by Dunn’s multiple comparisons test (for >2 groups). Statistical significance was set at *P*<0.05. Data analysis and visualization were performed using GraphPad Prism® version 5.0 (GraphPad Software, Inc., USA).

## 3 Results

### 3.1 Germ-free mice display anti-commensal hyperreactivity following priming with *E. coli ompC_gp61*

The genetically modified commensal *E. coli ompC_gp61* strain was initially developed to be able to study *E. coli* (gp61)-specific CD4 T cell responses (24). We have previously demonstrated that this model allows the tracking of *E. coli* (gp61)-specific CD4 T cell responses following systemic i.v. priming. Here, we investigated whether the genetic modification to insert the gp61 T cell epitope into loop 7 of OmpC, as described for microbial cell-surface display (36), has an impact on induction of mucosal versus systemic humoral *E. coli*-specific immunoglobulin (Ig) responses. Because the precursor frequency of gp61-specific CD4 T cells in naïve C57BL/6 mice is very low (37–39), we did not expect that addition of a single additional epitope to the *E. coli* genome would have a significant impact on the overall immunogenicity and therefore we tested this hypothesis.

**Figure 1:**
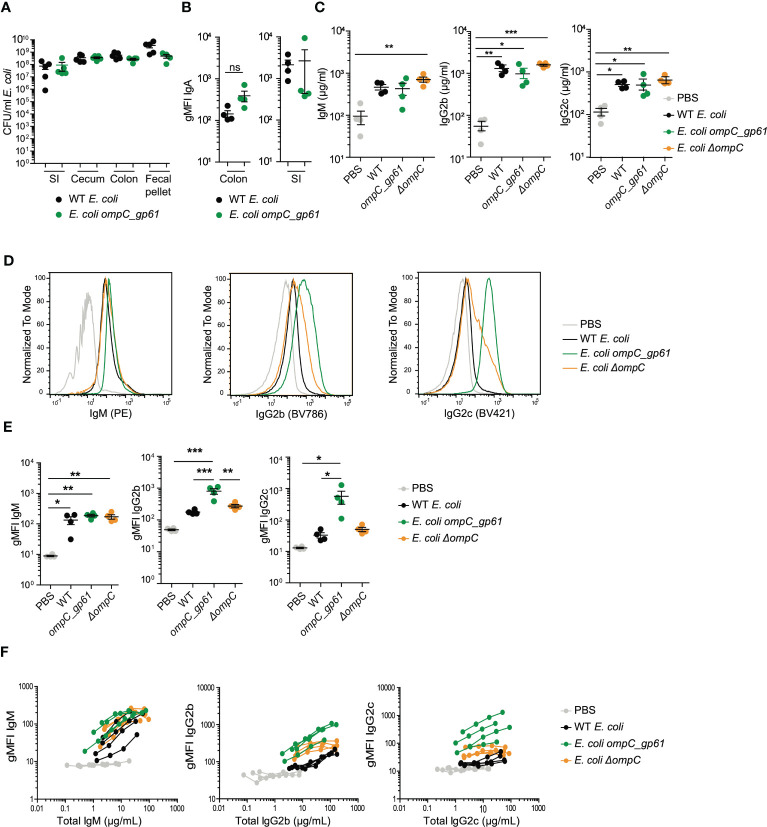
Hyperreactive anti-*E. coli* IgG responses following immunization with *E*. *coli_gp61*. **(A)** *E*. *coli* colonization levels in small intestine (SI), cecum, colon and in fecal pellets 14 days post systemic *E*. *coli* priming. Data representative of two independent experiments, error bars represent mean ± SEM. *P* values were calculated with a standard student t test or in case of significantly different variance between groups a non-parametric Kruskal Wallis with Dunn’s post-test. **(B)** *E*. *coli* specific IgA was assessed by bacterial flow cytometry in SI and colon content collected 14 days post systemic *E*. *coli* priming. Error bars represent mean ± SEM. **(C)** Total serum Ig titers 14 days post systemic priming with WT *E*. *coli*, *E*. *coli ompC_gp61* and *E*. *coli ΔompC*. Data representative of four independent experiments, error bars represent mean ± SEM. Representative plots **(D)** and gMFI quantification **(E)** of bacterial flow cytometric analysis of *E*. *coli* incubated with the indicated mouse sera (serum dilution 1:10, autologous incubation). Data representative of four independent experiments, error bars represent mean ± SEM. **(F)** *E*. *coli* bacterial flow cytometric analysis with serial dilution of serum (1:10, 1:30, 1:90 and 1:270, autologous incubation). Each line represents one mouse. The x-axis denotes total Ig concentration in the assay. Data representative of four independent experiments. ns: P > 0.05; *P ≤ 0.05; **P ≤0.01; ***P ≤0.001.

We first confirmed that both the parental wild-type *E. coli* MG1655 and the genetically modified *E. coli ompC_gp61* strain colonize germ-free mice to the same level. Germ-free C57BL/6 mice were colonized with pure cultures of either strain. Both strains equally colonized the small intestine (SI), cecum, colon, and fecal pellet to the same level (**Figure 1A**). We next measured the induction of *E. coli*-specific IgA in the colon and SI by measuring the amount of *E. coli* surface bound IgA using bacterial flow cytometry and found no significant difference in the induction of *E. coli*-specific IgA by the two strains (**Figure 1B**).

Next, we investigated the induction of total (non-specific) immunoglobulin responses following systemic priming. We tested this in germ-free mice in the absence of intestinal colonization with other bacteria and their metabolites. To do so, we systemically immunized germ-free female C57BL/6 mice with the parental WT, the *E. coli ompC_gp61*, or the ompC-deficient *E. coli ΔompC* strain, to control for potential effects mediated by a complete OmpC loss of function, by i.v. priming on (d0) and boosting on (d7) with 10^7^ CFU to obtain optimal Ig responses (**Figure S1A**).

Following i.v. immunization all three *E. coli* strains (WT, *ompC_gp61* and *ΔompC*) were cleared from the blood stream with identical kinetics (**Figure S1B**). Intravenous prime-boost immunization resulted in elevated total non-specific serum levels of IgM, IgG2b, and IgG2c compared to naïve germ-free levels (mice injected with PBS) with no difference in total (non-specific) serum Ig levels observed between the three immunized groups (**Figure 1C**). We next measured *E. coli*-surface specific Ig responses by bacterial flow cytometry (**Figure S1C**). Sera from mice immunized with the genetically modified *E. coli ompC_gp61* strain displayed significantly increased *E. coli*-specific IgG2b and IgG2c immunoreactivity compared to sera from WT or *E. coli ΔompC* immunized mice (**Figures 1D–F**). No differences in IgG1 or IgG3 reactivity were observed (data not shown). This indicated that the commensal *E. coli ompC_gp61* strain exhibited increased systemic immunogenicity compared to the parental WT *E. coli* or OmpC-deficient *E. coli ΔompC* strains.

### 3.2 Hyperreactive anti-commensal IgG responses are T cell-dependent and regulated by intestinal colonization

**Figure 2:**
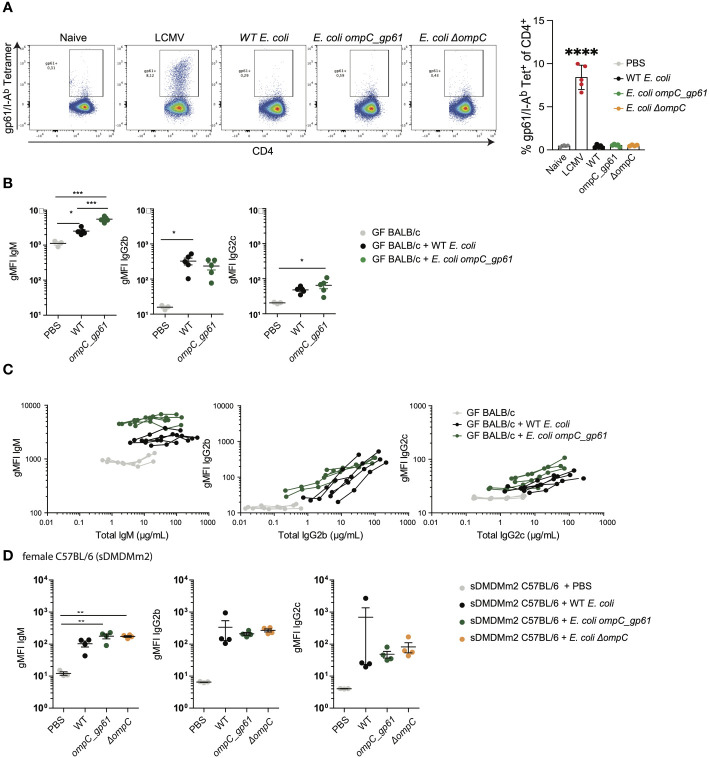
Anti-commensal IgG hyperreactivity is T cell-dependent and regulated by the microbiota. **(A)** Representative flow cytometry dot plots and quantification of gp61/I-A^b^ tetramer positive splenic CD4 T cells. Mice were i.v. primed (d0) and boosted (d7) with either of the three *E*. *coli* strains or were infected with 200pfu LCMV-Arm on d0. Gp61-specific CD4 T cell responses in the spleen were assessed on day 14. **(B)** gMFI quantification of bacterial flow cytometric analysis of *E*. *coli* incubated with the indicated sera of female germ-free BALB/c mice primed with either WT *E*. *coli* or *E*. *coli ompC_gp61*. Error bars represent mean ± SEM. **(C)** *E*. *coli* bacterial flow cytometry analysis with serial dilution of serum (1:10, 1:30, 1:90 and 1:270, autologous incubation) of germ-free BALB/c mice iv injected with the indicated *E*. *coli* strain. Each line represents one mouse. The x-axis denotes total Ig concentration in the assay. **(D)** gMFI quantification of bacterial flow cytometric analysis of *E*. *coli* incubated with the indicated sera of germ-free female sDMDMm2 colonized C57BL/6 mice i.v. injected with the indicated *E*. *coli* strain. Error bars represent mean ± SEM. *P ≤ 0.05; **P ≤0.01; ***P ≤0.001; ****P ≤ 0.0001.

One potential explanation for the observed increased immunogenicity could be that primed endogenous gp61-specific T cells significantly enhance class switch recombination of *E. coli*-specific B cells. Therefore, we measured gp61-specific CD4 T cells in the spleen by tetramer staining following priming with the three different *E. coli* strains (**Figure 2A**). As a positive control we used infection with 200pfu LCMV Armstrong. While LCMV, as expected, induced a strong gp61-specific systemic CD4 T cell response, we could not detect any gp61-specific CD4 T cells by tetramer staining following priming with either of the *E. coli* strains including *E. coli ompC_gp61* (**Figure 2A**) This is likely due to the very low frequency of gp61-specific CD4 T cells present in naïve C57BL/6 mice (37–39) and much weaker T cell priming by *E. coli* compared to a productive viral infection with a replicating virus. We therefore employed an alternative indirect approach to test whether the observed effect is dependent on the presence of an I-A^b^ molecule required for the presentation of the gp61 epitope. The same prime/boost protocol was applied to germ-free female BALB/c mice that harbour I-A^d^ and an I-E^d^ MHC class II molecules and do not possess the required I-A^b^ molecule to present gp61. In germ-free BALB/c mice we could no longer observe increased IgG immunoreactivity after priming with *E. coli ompC_gp61* (**Figures 2B, C**). Interestingly, we observed increased *E. coli*-specific IgM immunoreactivity following *E. coli ompC_gp61* priming of germ-free BALB/c mice, indicating the potential existence of a T cell-independent hyper-reactivity. To demonstrate T cell-dependence of the observed IgG effect we subjected germ-free female T-cell deficient *Tcrbd^-/-^* C57BL/6 mice to the same prime-boost protocol with WT *E. coli* or *E. coli ompC_gp61* and measured *E. coli*-specific reactivity in the serum one week after the boost. We found that the induction of *E. coli*-specific IgG2b and IgG2c was T cell-dependent and, therefore, increased IgG reactivity was no longer observed in the absence of T cell help. On the other hand, IgM immunoreactivity was still observed further indicating an additional potential T cell-independent mechanism (**Figure S2A**).

We next investigated how intestinal colonization after birth impacts on the observed anti-commensal hyperreactivity. We used female C57BL/6 mice colonized with the stable defined moderately diverse gnotobiotic intestinal microbiota (sDMDMm2) (25, 26) and subjected the mice to the same prime/boost protocol using the three *E. coli* strains. In sDMDMm2 colonized female C57BL/6 mice hyperreactive *E. coli*-specific IgG responses following priming with *E. coli ompC_gp61* were no longer observed (**Figure 2D**). Furthermore, hyperreactive *E. coli*-specific IgG responses were only observed in germ-free female but not in germ-free male C57BL/6 mice (**Figure S2B**). In germ-free C57BL/6 males, *E. coli ompC_gp61* priming resulted in increased *E. coli*-specific IgM immunoreactivity but not increased *E. coli*-specific IgG immunoreactivity (**Figure S2B**), similar to what we observed in germ-free BALB/c mice (**Figure 2A**). Such sex differences in the adaptive immune response have also previously been described (40, 41). Therefore, we performed all subsequent experiments in germ-free female C57BL/6 mice to investigate the observed increased IgG immunoreactivity following priming with *E. coli ompC_gp61*.

Taken together, these findings indicate that the systemic immune system of germ-free female mice is sensitive enough to detect the addition of a single T helper epitope to a commensal *E. coli* strain resulting in a hyperreactive *E. coli*-specific IgG response. Intestinal colonization abrogates this systemic anti-commensal hyperreactivity indicating that intestinal colonization not only induces local mucosal regulation of anti-commensal responses but also regulates systemic anti-commensal hyperreactivity.

### 3.3 Consequences of OmpC modification on bacterial surface structure, OmpC function, and bacterial metabolism

**Figure 3:**
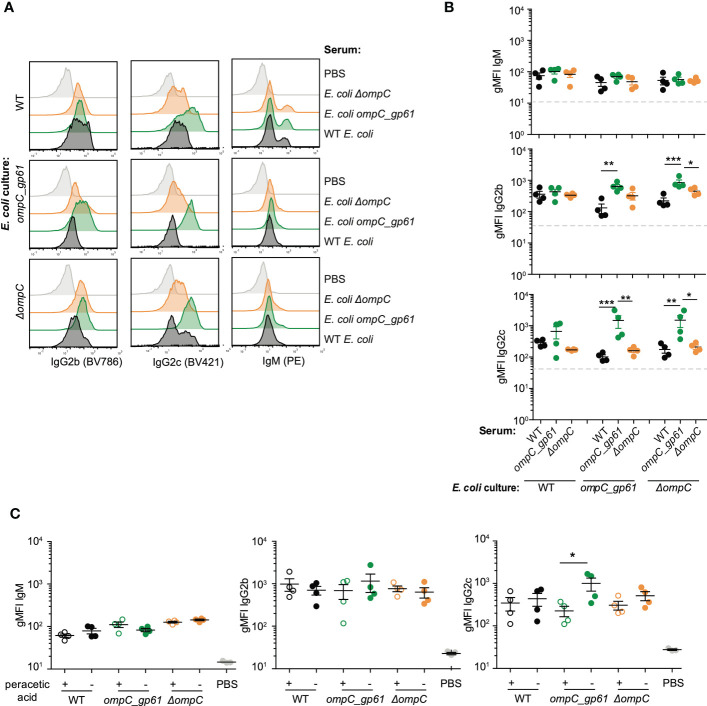
Cross-reactivity between the three MG1655 *E*. *coli* strains. Representative plots **(A)** and gMFI quantification **(B)** of bacterial flow cytometric analysis of *E*. *coli* incubated with the indicated mouse sera (serum dilution 1:10). Dashed line indicates *E*. *coli* incubated with sera of germ-free mice. Data representative of three independent experiments, error bars represent mean ± SEM. **(C)** gMFI quantification of bacterial flow cytometric analysis of IgM, IgG2b and IgG2c of *E*. *coli* (live or peracetic acid treated) incubated with the indicated mouse sera (serum dilution 1:10, autologous incubation). Error bars represent mean ± SEM. ns: P > 0.05; *P ≤ 0.05; **P ≤0.01; ***P ≤0.001.

An alternative hypothesis for the observed increased immunogenicity of *E. coli ompC_gp61* is that this is mediated by either structural or metabolic changes in *E. coli ompC_gp61*. To test whether increased immunogenicity was mediated by structural changes in the bacterial surface of the *E. coli ompC_gp61* strain, we incubated serum samples from female germ-free C57BL/6 mice primed with either of the three *E. coli* strains (WT, *ompC_gp61*, and *ΔompC*) not just with the autologous strain that was used for priming but also with the other two strains to check for cross-reactivity. We found that IgG2b and IgG2c from sera of *E. coli ompC_gp61* immunized mice displayed increased *E. coli* surface binding to the autologous *E. coli ompC_gp61* as well as the *E. coli ΔompC* strain (**Figures 3A, B**). Intriguingly, we did not observe increased IgG2b reactivity and increased *E. coli*-specific IgG2c reactivity did not reach statistical significance when *E. coli ompC_gp61* primed serum was tested against the WT *E. coli* strain (**Figure 3B**). It is possible that modification of ompC in *E. coli ompC_gp61* and deletion of ompC in *E. coli ΔompC* might have introduced changes in the *E. coli* surface structure that allow for better accessibility of immunoglobulins to antigens. Nevertheless, serum from *E. coli ompC_gp61* primed mice still exhibited increased *E. coli*-specific reactivity when tested against *E. coli ompC_gp61* or *E. coli* ΔompC compared to sera from mice primed with either WT *E. coli* or *E. coli* ΔompC (**Figure 3B**).

In addition, the IgM, IgG2b, and IgG2c responses induced by all three *E. coli* strains did not display cross-reactivity with other bacterial species as no significant binding to species such as *P. aerginosa, S. typhimurium, C. rodentium, K. oxytoca* or *A. muciniphila* was observed (**Figures S3A, B**). There was also no significant cross-reactivity with the *E. coli* strain Xen14, confirming that the Igs induced by the three *E. coli* strains are very specific for the MG1655 strain background (**Figure S3**).

We next investigated whether increased immunogenicity of *E. coli ompC_gp61* is mediated by changes in the presence or abundance of *E. coli*-derived metabolites. First, we tested whether the observed increased immunogenicity required immunization with live bacteria. We performed prime-boost immunizations of germ-free female C57BL/6 mice with either live *E. coli* strains, as before, or peracetic acid killed and fixed *E. coli* strains. Peracetic acid fixation kills bacteria but maintains bacterial surface and antigen integrity (42).

We found that while peracetic acid killed/fixed *E. coli* strains still induced *E. coli*-specific IgM, IgG2b, and IgG2c responses, *E. coli*-specific IgG hyperreactivity following priming with *E. coli ompC_gp61* was no longer observed (**Figure 3C**). We concluded that the observed hyperreactivity required immunization with live bacteria with an active metabolism.

We next measured the metabolite profiles of the WT *E. coli*, *E. coli ompC_gp61*, and *E. coli ΔompC* strains. Each of the three *E. coli* strains was grown in liquid cultures using the same LB media that was used to grow bacteria for i.v. injections. Both, the culture supernatant as well as the bacterial pellet were then subjected to metabolomic analysis by liquid chromatography with tandem mass spectroscopy including standards to identify peaks to measure changes in the presence and abundance of secreted and intracellular metabolites.

**Figure 4:**
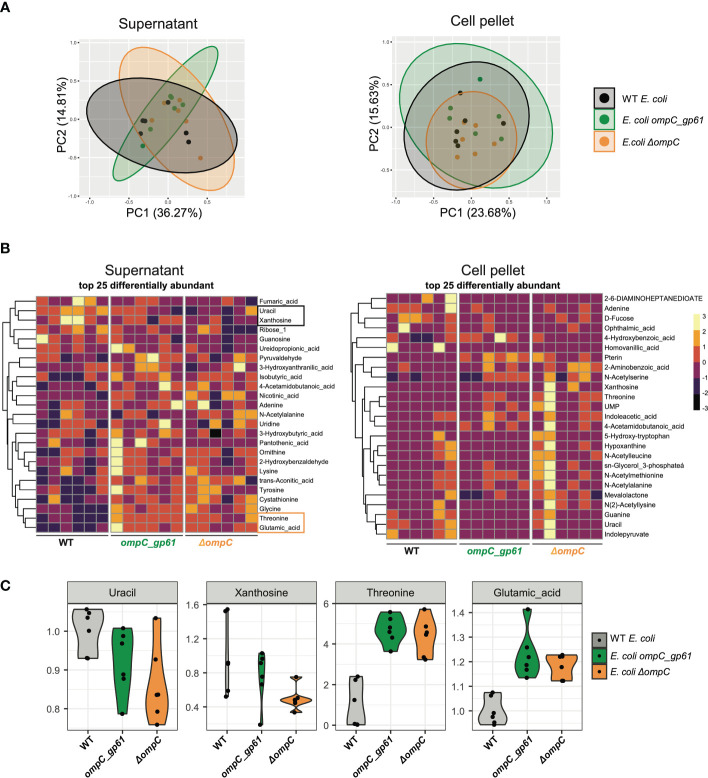
Impact of OmpC modification on metabolomic profiles of secreted and intracellular metabolites. **(A)** Principal component plots showing the 2 axes that explain most variation in the metabolite profiles of the supernatant and cell pellets of WT *E*. *coli*, *E*. *coli ompC_gp61* and *E*. *coli Δ*ompC (supernatant: P = 0.29, cell pellet: P=0.61) **(B)** Heatmap of the 25 most differentially abundant metabolites based on strain in the supernatant and cell pellet. **(C)** Violin plots for the abundance of specific metabolites. One representative of three independent experiments with similar results is shown.

Principal component analysis of all detected secreted (supernatant) and intracellular (pellet) metabolites did not reveal any significant metabolic differences between the three strains (**Figure 4A**). To assess changes in more detail, we generated heatmaps of the 25 metabolites that were most differentially abundant to identify potential metabolites that may be produced or secreted differentially in the three *E. coli* strains and could contribute to the observed altered immunogenicity (**Figure 4B**). The heatmaps revealed that the abundance profile of the 25 most differentially abundant metabolites in *E. coli ompC_gp61* was closer to the OmpC-deficient strain for the secreted metabolites while the three profiles looked similar for all three strains for the intracellular metabolites (**Figure 4B**). Even at the level of individual metabolites, we could not detect significant differences between *E. coli ompC_gp61* and *E. coli* Δ*ompC* (**Figure 4C**). Since anti-commensal hyperreactivity was only observed with *E. coli ompC_gp61* and not *E. coli* Δ*ompC*, it is therefore unlikely that metabolic changes contributed to the observed effect. However, these findings show that OmpC function in *E. coli ompC_gp61* is compromised. Because OmpC is known to be involved in controlling bacterial cell wall/membrane permeability due to its function as a passive diffusion channel, we also investigated whether the alteration of OmpC in *E. coli ompC_gp61* affected bacterial membrane permeability. Altered membrane integrity can be detected by measuring the entry of membrane impermeable molecules such as SYTOX Green (43) or SYTO BC, a cell permeable dye, into the cell (44).

**Figure 5:**
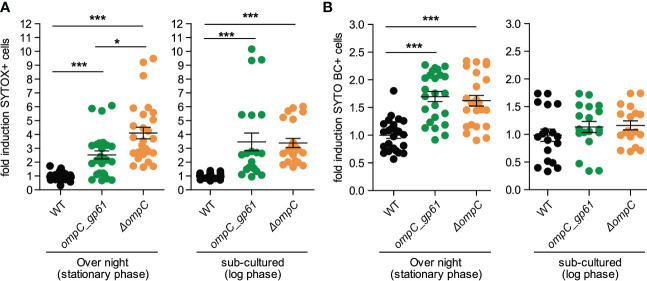
Effects of OmpC modification on membrane integrity. Fold induction of **(A)** SYTOX Green and **(B)** SYTO BC positive cells as assessed by bacterial flow cytometry of *E coli* stained at stationary phase and log-phase. Pooled data from four independent experiments. Error bars represent mean ± SEM. *P ≤ 0.05; ***P ≤0.001.

We cultured the three different *E. coli* strains under the same conditions that were used for preparing the bacteria for i.v. injections and then performed a permeability assay using SYTOX and SYTO BC. Like for the measured metabolite profiles, *E. coli ompC_gp61* displayed similar membrane permeability defect as *E. coli* Δ*ompC* and both showed increased permeability compared to the parental WT strain (**Figures 5A, B**). However, increased membrane permeability alone does not explain the observed increased immunogenicity of *E. coli ompC_gp61* because *E. coli* Δ*ompC* did not display increased immunogenicity (**Figures 1E, F**). Based on these findings, the *E. coli* Δ*ompC* strain is the more appropriate experimental control than the WT *E. coli* strain for the presented experiments.

Taken together, our findings demonstrate that germ-free female mice display a hypersensitive systemic immune system that is able to detect addition of a single epitope to *E. coli* responding with hyperreactive *E. coli*-specific IgG responses that are T cell-dependent. Intestinal colonization regulates this systemic anti-commensal hyperreactivity.

## 4 Discussion

It is well described that intestinal colonization induces local mucosal immune regulation to limit anti-commensal reactivity (1). Whether intestinal colonization also regulates systemic anti-commensal reactivity remained unclear. It is now well accepted and demonstrated that intestinal microbiota-derived metabolites modulate local as well as systemic immune responses (1). For example, short chain fatty acids (SCFA) such as butyrate or acetate promote local regulatory T cell induction and microbiota-derived inosine has been shown to be able to promote systemic anti-tumor responses (1, 23). Most of the studies investigating the crosstalk between intestinal commensal bacteria and the immune system and the role of bacterial metabolites have focused on commensal bacteria residing and contained within the intestinal lumen. It is well known, however, that the intestinal barrier can be temporarily compromised leading to increased permeability resulting in repeated systemic translocation of intestinal bacteria causing systemic immune priming including, in addition to rapid innate immune responses, also systemic adaptive immune responses. How commensal-derived metabolites modulate systemic adaptive anti-commensal immune responses is less well studied.

Although the gp61-specific CD4 T cell precursor frequency is very low (37–39), we found that addition of the gp61epitope to a commensal *E. coli* resulted in increased *E. coli*-specific IgG immunoreactivity. However, this was only observed in germ-free female C57BL/6 mice and hyperreactivity was abrogated in mice that were colonized at birth.

It is known that males and females differ in their immune responses to foreign and self-antigens (40), which includes vaccine responses (45) and anti-viral responses (46, 47). While sex differences in intestinal microbiome composition and function have also been described (48, 49), microbiome effects can be excluded in our study as sex differences were observed in the immune responses of germ-free mice.

Because anti-commensal hyperreactivity was only observed following priming with live bacteria, we also investigated whether differential metabolic profiles potentially also contribute to systemic anti-commensal hyperreactivity. We show that the OmpC modification to introduce the gp61 epitope generates a loss-of-function phenotype although the protein is still expressed, processed, and gp61 is presented (24). OmpC and OmpF are major passive diffusion channels for small hydrophilic molecules in the outer membrane of *E. coli* (50). Furthermore, it has been shown that porins play an important role in the maintenance of membrane integrity (51) and deletion of these molecules has been associated with resistance to antibiotics (52). *E. coli* anti-OmpC antibodies have been found in sera of Crohn’s disease patients and their families as well as healthy individuals, indicating that OmpC is a dominant immunogen (13). Of note, OmpC-specific antibodies-initiated antibody-dependent classical pathway for the clearance of *E. coli* and loss of OmpC contributes to escaping bactericidal activity (53). The immunogenicity of OmpC was also investigated in mice, Liu et al. demonstrated that the recombinant OmpC and OmpF proteins from *E. coli* (ExPEC) stimulated strong IgG1 and IgG2a responses (54). Therefore, we also investigated whether insertion of gp61 into loop 7 of OmpC had an impact on bacterial surface structure or OmpC function as a passive diffusion channel. We found that the, compared to the *E. coli ompC_gp61* and the *E. coli ΔompC* strain, the WT *E. coli* strain displayed reduced binding to hyperreactive serum from *E. coli ompC_gp61* primed mice, indicating a possible reduction in surface antigen accessibility.

Hence, our findings indicate that the systemic immune system of germ-free female mice displays increased sensitivity allowing for the detection of a single additional T cell epitope resulting in a hyperreactive anti-commensal IgG response, which is regulated and abrogated in mice colonized at birth.

Zeng et al. have previously demonstrated that systemic IgG induced by a complex intestinal microbiota is cross-reactive enough to protect from systemic infection by other symbiotic or even pathogenic bacteria (4). While we found no evidence for cross-reactivity of IgG induced by *E. coli* in our experiments, it is important to realize that our experiments were performed by immunizing germ-free mice, indicating that the protective IgG described by Zeng et al. likely requires the presence of a complex microbiota.

We further show that anti-commensal hyperreactivity is not directed against the OmpC protein specifically but rather against the bacterial cell surface in general or maybe against another dominant antigen found in the bacterial cell wall. Zeng et al. have identified Murein Lipoprotein (MLP) as a major microbiota-derived antigen inducing steady-state IgG responses in mice colonized with a complex microbiota (4). MLP is a major component of the outer membrane of gram-negative enteric bacteria, and it is therefore likely the observed increased immunogenicity in our study is, at least partially, also directed against MLP in our study.

The outer membrane of gram-negative bacteria controls the cellular uptake of beneficial as well as toxic compounds such as bile acid, detergents, and antibiotics. OmpC and OmpF, the major outer membrane proteins in *E. coli*, allow the diffusion of small hydrophilic molecules, including nutrients and antibiotics (52, 55). Mutations and deletions of these porins have been associated with antibiotics resistance (51, 52). Moreover, altered transport of small molecules and antibiotics across the outer membrane have been described in OmpC mutants isolated from clinical strains of multi-drug resistant *E. coli* (56). Besides their role as channels, porins also contribute to membrane stability (52). For example, ompC forms a complex with the lipoprotein MlaA, this complex is critical for the maintenance of the outer membrane lipid asymmetry in *E. coli* (57). Accordingly, cells lacking ompC display permeability defects (51, 57) and subsequently enhanced sensitivity to envelop stress, including SDS/EDTA (57) and salt stress (51, 58). Interestingly, high salt concentration have previously shown to modulate OmpC expression (59) and increase the permeability for β-lactams and lactose through OmpC in *E. coli* (58).

In conclusion, intestinal colonization also regulates systemic anti-commensal hyperreactivity. Future studies will investigate whether the same or different metabolites/factors that promote local mucosal regulation are involved in systemic regulation.

## Data availability statement

The metabolomics data in this study is available at the NIH Common Fund's National Metabolomics Data Repository (NMDR) website, the Metabolomics Workbench, https://www.metabolomicsworkbench.org, Project ID PR001467. The data can be accessed directly via it's Project DOI: 10.21228/M83H68.

## Ethics statement

The animal study was reviewed and approved by University of Calgary Health Sciences Animal Care Committee.

## Author contributions

RB, MK, KB, KW, LM, AZ, CT, RN, IS, and SA performed experiments and analyzed data. BM provided reagents, helped design experiments, and discussed data. RB and MBG wrote the manuscript. All authors contributed to the article and approved the submitted version.

## Supplementary material

Supplementary Figure 1
**(A)** Experimental setup for the prime boost experiments. **(B)** Bacteraemia levels in the blood following intravenous injection of 10^7^ CFU of the indicated *E. coli* strain into germ-free female C57BL/6 mice measured by aerobic plating on LB agar plates. Error bars represent mean ± SEM. **(C)** Gating strategy for bacterial flow cytometric analysis.

Supplementary Figure 2 Increased systemic immunogenicity is T cell- and sex-dependent. **(A)** *E. coli* bacterial flow cytometry analysis with serial dilution of serum (1:10, 1:30, and 1:90, autologous incubation) of female germ-free *TCRbd^-/-^* mice iv injected with the indicated *E. coli* strain. Each line represents one mouse. The x-axis denotes total Ig concentration in the assay. Data representative of two independent experiments **(B)** gMFI quantification of bacterial flow cytometric analysis of *E. coli* incubated with the indicated sera (serum dilution 1:10, autologous incubation) of male C57BL/6 mice. Error bars represent mean ± SEM.

Supplementary Figure 3
*E. coli*-induced Ig responses are not species cross-reactive. gMFI quantification of bacterial flow cytometric analysis of **(A)** IgG2b and **(B)** IgG2c of bacteria incubated with the indicated mouse sera (serum dilution 1:30). Blue bars indicate background fluorescence (samples stained with fluorescently labelled secondary antibody only). Data representative of two independent experiments, error bars represent mean ± SEM.
