# Supplementary material for: Intestinal colonization regulates systemic anti-commensal immune sensitivity and hyperreactivity
Source: Front Immunol. 2023 May 22;14:1030395. doi: 10.3389/fimmu.2023.1030395 (PMC10239946; doi:10.3389/fimmu.2023.1030395)
Supplement: Supplementary Figure 1 — (A) Experimental setup for the prime boost experiments. (B) Bacteraemia levels in the blood following intravenous injection of 107 CFU of the indicated E. coli strain into germ-free female C57BL/6 mice measured by aerobic plating on LB agar plates. Error bars represent mean ± SEM. (C) Gating strategy for bacterial flow cytometric analysis. [file Image_1.pdf]

Supplemental Figures

Front. Immunol. 14:1030395.

doi: 10.3389/fimmu.2023.1030395

Intestinal colonization regulates systemic anti-commensal immune sensitivity and hyperreactivity

Regula Burkhard[1], Mia Koegler[1], Kirsty Brown[2], Kirsten Wilson[1], Lukas F. Mager[2], Amanda Z. Zucoloto[2], Carolyn Thomson[2], Roopa Hebbandi Nanjundappa[1], Isla Skalosky[1], Shokouh Ahmadi[1], Braedon McDonald[3,4,5] and Markus B. Geuking[1,3,4]

[1]Department of Microbiology, Immunology and Infectious Diseases, Cumming School of Medicine, University of Calgary, Calgary, AB, Canada,
[2]Department of Physiology and Pharmacology, Cumming School of Medicine, University of Calgary, Calgary, AB, Canada,
[3]Snyder Institute of Chronic Diseases, Cumming School of Medicine, University of Calgary, Calgary, AB, Canada,
[4]Immunology Research Group, Cumming School of Medicine, University of Calgary, Calgary, AB, Canada,
[5]Department of Critical Care Medicine, Cumming School of Medicine, University of Calgary, Calgary, AB, Canada

Supplemental Figure 1

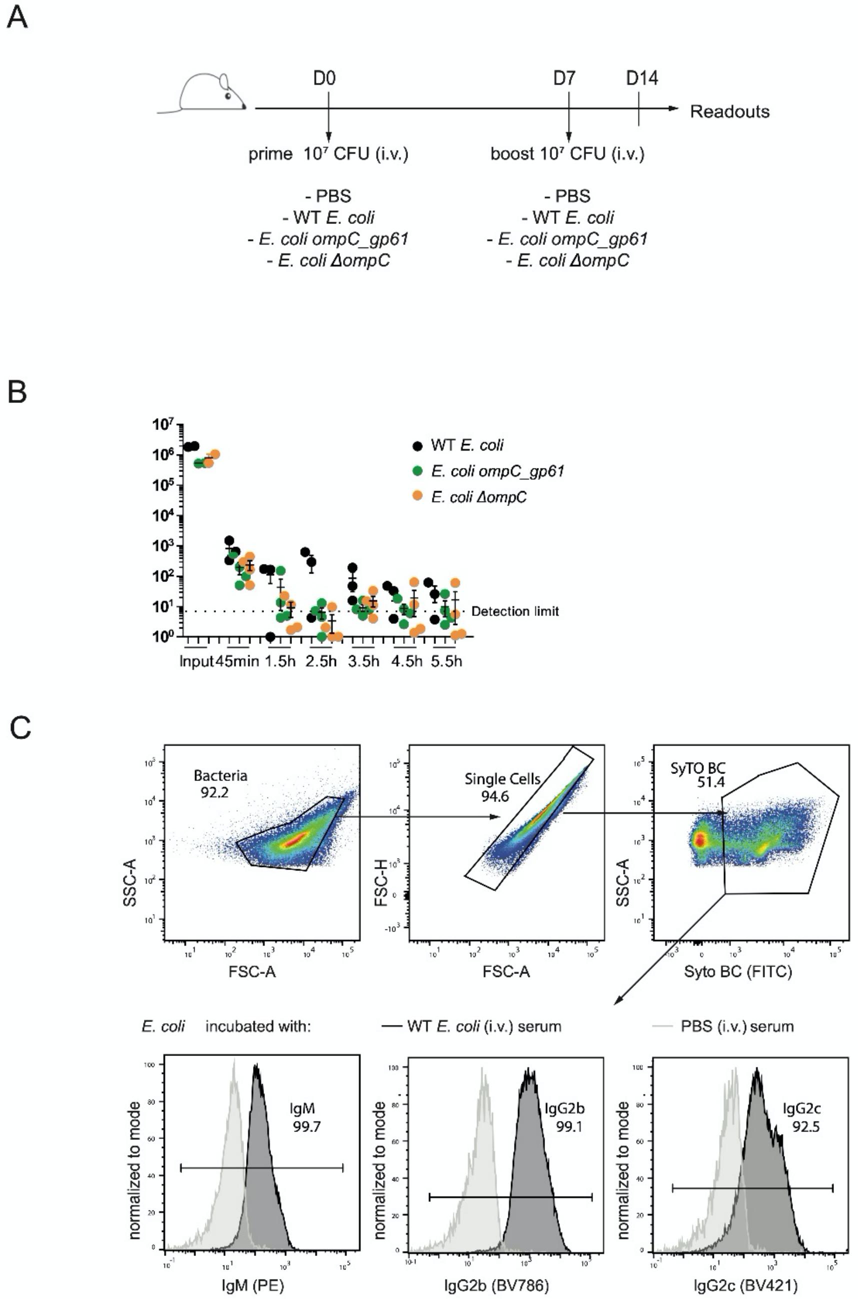

Supplemental Figure 1. (A) Experimental setup for the prime boost experiments. (B) Bacteraemia levels in the blood following intravenous injection of 10^7 CFU of the indicated *E. coli* strain into germ-free female C57BL/6 mice measured by aerobic plating on LB agar plates. Error bars represent mean ± SEM. (C) Gating strategy for bacterial flow cytometric analysis.

Supplemental Figure 2

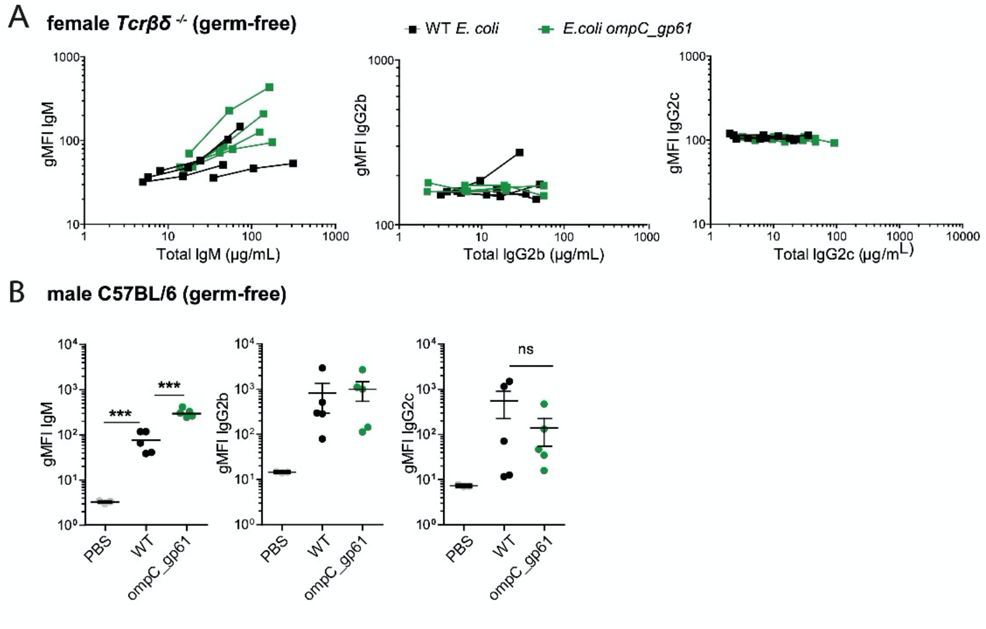

Supplemental Figure 2. Increased systemic immunogenicity is T cell- and sex-dependent. (A) *E. coli* bacterial flow cytometry analysis with serial dilution of serum (1:10, 1:30, and 1:90, autologous incubation) of female germ-free *TCRbd*$^{-/-}$ mice iv injected with the indicated *E. coli* strain. Each line represents one mouse. The x-axis denotes total Ig concentration in the assay. Data representative of two independent experiments (B) gMFI quantification of bacterial flow cytometric analysis of *E. coli* incubated with the indicated sera (serum dilution 1:10, autologous incubation) of male C57BL/6 mice. Error bars represent mean ± SEM. **P* ≤ 0.05; ***P* ≤0.01; ****P* ≤0.001.

Supplemental Figure 3

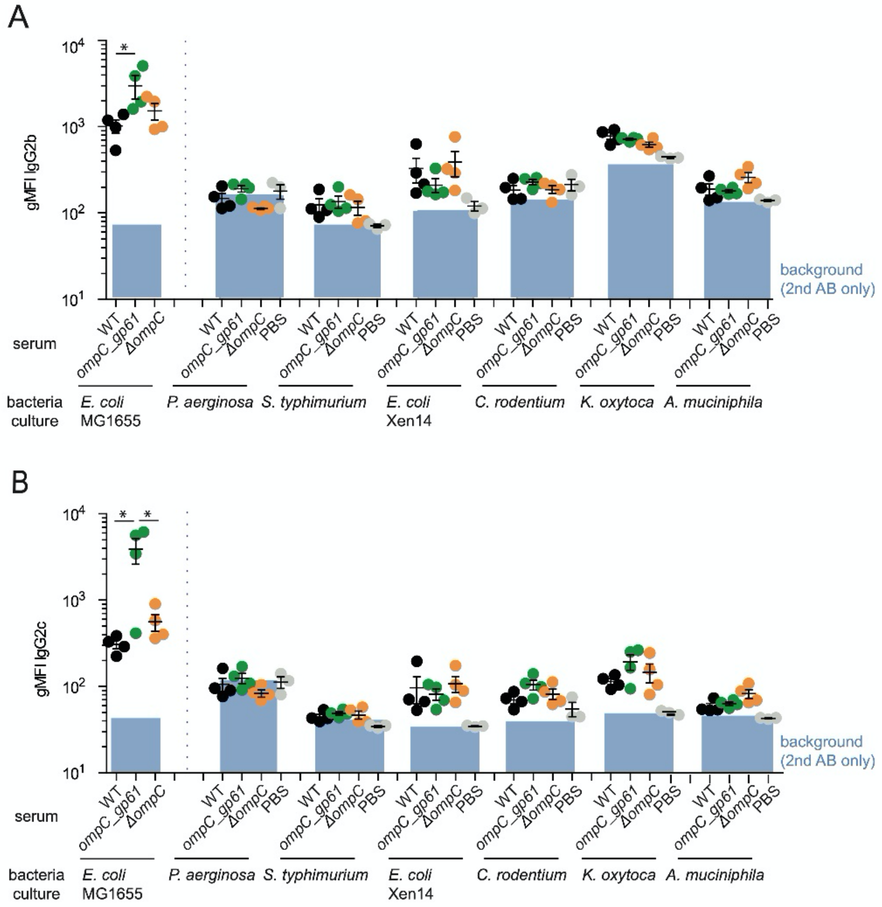

Supplemental Figure 3. ***E. coli*-induced Ig responses are not species cross-reactive.** gMFI quantification of bacterial flow cytometric analysis of (A) IgG2b and (B) IgG2c of bacteria incubated with the indicated mouse sera (serum dilution 1:30). Blue bars indicate background fluorescence (samples stained with fluorescently labelled secondary antibody only). Data representative of two independent experiments, error bars represent mean ± SEM. **P* ≤ 0.05; ***P* ≤0.01; ****P* ≤0.001.
